# Ketone Bodies Induce Unique Inhibition of Tumor Cell Proliferation and Enhance the Efficacy of Anti-Cancer Agents

**DOI:** 10.3390/biomedicines11092515

**Published:** 2023-09-12

**Authors:** Anna I. Miller, David Diaz, Bo Lin, Patryk K. Krzesaj, Sarah Ustoyev, Alfred Shim, Eugene J. Fine, Ehsan Sarafraz-Yazdi, Matthew R. Pincus, Richard D. Feinman

**Affiliations:** 1 Department of Cell Biology, SUNY Downstate Health Sciences University, 450 Clarkson Avenue, Brooklyn, NY 11203, USA; 2 Department of Pathology, SUNY Downstate Health Sciences University, 450 Clarkson Avenue, Brooklyn, NY 11203, USA; 3 Department of Pathology, AdventHealth, 301 Memorial Medical Pkwy, Daytona Beach, FL 32117, USA; 4 Department of Radiology (Nuclear Medicine), Albert Einstein College of Medicine, New York, NY 10461, USA; 5 NomoCan Pharmaceuticals LLC, New York Blood Center, 310 East 67th Street, New York, NY 10065, USA

**Keywords:** ketone bodies, chemotherapeutic agents, cancer cell viability, IC_50_ values

## Abstract

The ketone bodies, sodium and lithium salts of acetoacetate (AcAc) and sodium 3-hydroxybutyrate (3-HB; commonly called beta-hydroxybutyrate) have been found to inhibit the proliferation of cancer cells. Previous studies have suggested that lithium itself may be an inhibiting agent but may be additive or synergistic with the effect of AcAc. We previously found that sodium acetoacetate (NaAcAc) inhibits the growth of human colon cancer cell line SW480. We report here similar results for several other cancer cell lines including ovarian, cervical and breast cancers. We found that NaAcAc does not kill cancer cells but rather blocks their proliferation. Similar inhibition of growth was seen in the effect of lithium ion alone (as LiCl). The effect of LiAcAc appears to be due to the combined effects of acetoacetate and the lithium ion. The ketone bodies, when given together with chemotherapeutic agents, rapamycin, methotrexate and the new peptide anti-cancer agent, PNC-27, substantially lowers their IC_50_ values for cancer cell, killing suggesting that ketone bodies and ketogenic diets may be powerful adjunct agents in treating human cancers.

## 1. Introduction

Over the past decade, increasing attention in the treatment of cancer has been focused on metabolic control of cancer cell proliferation [1]. An important aspect of this approach has been the use of low carbohydrate and ketogenic diets. Such diets have been found to induce diminution of in vivo cell proliferation of tumors, attributed at least in part, to the increased presence of the ketone bodies, acetoacetate and 3-hydroxybutyrate [2,3,4,5,6,7,8]. Mice with syngeneic breast cancers treated with chemotherapeutic agents were found to survive for significantly longer periods if they had been placed on ketogenic diets, rather than the control, non-ketogenic diet [8]. In corresponding in vitro studies, ketone bodies were found to block the growth of a variety of cancer cells in culture, while not affecting the growth of normal or untransformed cells [7].

These latter studies [7] showed that, at 10 mM acetoacetate, cancer cell proliferation was significantly reduced, with associated decreases in ATP production and, concomitantly, over-expression of uncoupling protein-2 (UCP-2), while untransformed cells were unaffected by this agent. A guiding principle in the emphasis on metabolism has been the Warburg effect [9], which holds that cancer cells primarily use glycolysis in preference to the Krebs cycle, even if oxygen is present.

Lithium salt is the commercially available form of acetoacetate, but it is known that lithium ions alone are capable of inhibiting proliferation of some cancer cells [10]. The effect of non-keto acid salts, such as lithium chloride, on cancer cell proliferation have been investigated [11,12]. Lithium chloride was found to reproduce the effect of lithium acetoacetate on an equimolar basis (at concentrations of 10–15 mM) on a number of different human cancer cell lines [11,12]. These included human breast cancer cell lines (MCF-7, MDA-MB-231 and Hs578T), SV40-large T antigen-immortalized non-transformed mammary luminal epithelial cells (HB2) and a normal human fibroblast cell line (HSF064) [1]. Of note, both agents were found to inhibit the viability of the two untransformed cell lines’ (HB2 and HSF064) growth to about the same extent as they did for the cancer cells [11]. Similar results were found in a study of neuroblastoma and renal cell carcinoma cell lines and two untransformed cell lines [2]. In these studies, sodium acetoacetate at the concentrations of the lithium compounds (10–15 mM) [11,12] did not have an inhibitory effect on cancer cells. In contrast, Fine, et al. [7] found that proliferation of multiple different human cancer cell lines was inhibited by 10 mM lithium acetoacetate while normal cells were unaffected by this treatment. Similarly, we determined the effect of sodium acetoacetate on the growth of a human colon cancer line (SW480) and a normal fibroblast cell line (HSF2617) [13]. Here, sodium acetoacetate inhibited cancer cell proliferation at concentrations between 30–60 mM while having no effect on normal cell growth [13] even at the highest concentration used (60 mM). In the studies of the ketogenic diet-treated syngeneic tumor-bearing mice [8], ketone bodies are endogenous and the question of the counterion does not arise.

Differences were likely due in part to concentration effects. Most of the studies performed on lithium acetoacetate and lithium chloride were in the 10 mM concentration range at which the sodium salt has little effect. With sodium acetoacetate and 3-hydroxybuyrate, inhibition of cancer cell growth was predominantly in the 30–60 mM range. On the other hand, recent findings [11,12] on the effects of lithium acetoacetate and lithium chloride on cancer and untransformed cells differed from the results of the prior study [7] which found that lithium acetoacetate was a strong inhibitor of cancer cell proliferation but did not affect normal cells. The findings suggest that lithium ion is non-discriminating in growth inhibition of cancer and normal cells.

Based on our findings that ketogenic diets enhance the effects of chemotherapeutic agents in treating syngeneic tumors in vivo and that acetoacetate and 3-hydroxybutyrate selectively inhibit tumor cell growth in cell culture [8], we surmised that these latter two agents would enhance the effects of chemotherapeutic agents in vitro. To this end, we found that sodium acetoacetate strongly affected the IC_50_ of rapamycin-induced SW480 colon cancer cell death such that the IC_50_ was reduced by more than 30-fold at levels of ketone body >40 mM [13].

We have now extended the studies with the SW480 line to a variety of other human tumors, including human breast cancer (MCF-7), ovarian cancer (SKOV-3) and squamous cell cervical cancer (HTB-35). We found that 3-HB and both LiAcAc and NaAcAc block cancer cell proliferation but are not cytotoxic to these cells. As expected, these agents were without effect on untransformed cells. (Ketone bodies are fuels for normal cells). In addition, we found that LiAcAc significantly lowers the IC_50_ of chemotherapeutic agents when killing cancer cells.

## 2. Materials and Methods

### 2.1. Cell Lines and Cultures

SW480 human colon cancer, MCF-7 human breast cancer, SKOV3 human ovarian cancer, HTB-35 human squamous cervical cancer cells and untransformed human HSF2617 and HFF fibroblasts were obtained from the ATCC (Manassas, VA, USA). All cell lines were cultured in DMEM at 37 °C and 5% CO_2_ pressure. Medium was supplemented with 10% fetal bovine serum, 4 mM glutamine, 1 mM pyruvate, 10 and 25 mM glucose, and 1% (*v*/*v*) penicillin and streptomycin in bicarbonate buffer at pH 7.2. Cell culture reagents were obtained from Corning through ThermoFisher (Fairlawn, NJ, USA).

### 2.2. Chemicals and Drugs

Sodium DL-3-hydroxybutyrate and cell culture grade dimethyl sulfoxide (DMSO) were purchased from MilliporeSigma (Burlington, MA, USA). Crystal violet and rapamycin were purchased from Alfa Aesar Chemicals of ThermoFisher. A stock of 1 mg/mL rapamycin was prepared by solution in sterile cell culture grade DMSO and maintained at −80 °C. The final DMSO concentration was maintained at 0.5% (*v*/*v*) either in the control or the treatment samples in all experiments. Sodium acetoacetate was prepared by reaction of equimolar ethyl acetoacetate and NaOH for 12 h at 4 °C [14]. Acetoacetate concentration was determined by a colorimetric method [15]. Methotrexate was obtained from Sigma Aldrich (St. Louis, MO, USA) and was dissolved in sterile distilled water and used directly as indicated in the Results section. The anti-cancer peptide, PNC-27, sequence H-Pro-Pro-Leu-Ser-Gln-Glu-Thr-Phe-Ser-Asp-Leu-Trp-Lys-Leu-Leu-Lys-Lys-Trp-Lys-Met-Arg-Arg-Asn-Gln-Phe-Trp-Val-Lys-Val-Gln-Arg-Gly-OH [16] was obtained from Biopeptides (San Diego, CA, USA); a stock solution of 1 mg/mL was prepared. Appropriate dilutions in triple distilled water were prepared for dose–response analysis experiments. Each diluted solution was subjected to sonication just prior to use. The concentration range employed for this peptide was 0–250 μg/mL.

### 2.3. Cell Treatment and Cell Proliferation Assay

Cells were grown to 80% confluence and detached with trypsin-EDTA 1X solution. Experimental cells were either incubated alone untreated or together with LiAcAc at concentrations ranging from 10–20 mM and NaAcAc ranging from 0–60 mM for 96 h except where indicated. In dose–response experiments in which the cells were incubated with chemotherapeutic compounds, i.e., rapamycin, methotrexate and PNC-27, dose–response curves were generated for each of these compounds at different concentrations of either LiAcAc or NaAcAc in 10 mM glucose DMEM/low glucose in 96 well tissue culture plates, along with controls without any additives. The crystal violet proliferation assay [17] was used to determine viable cells in all treatment conditions. Assays were measured on a Perkin–Elmer (Waltham, MA, USA) Victor3 multilabel plate reader. The trypan blue assay was employed to determine the number of non-viable cells as described previously [18].

Inhibition was considered to be significant if the *p*-value for the difference in the mean growth percentages at two different concentrations of KB was <0.05. For SW480 cells, all levels of inhibition for comparisons of means with that of the control were statistically significantly different for both KB agents. For MCF-7 cells, with NaAcAc, only cell growth percent for the 10 mM value compared with that for the control had a *p*-value > 0.05. Statistically significant differences in % growth for successive comparisons were obtained with 3-HB at all concentrations of this agent. For SKOV-3 cells incubated with NaAcAc, mean percent growth at 10 mM and 20 mM was not statistically different from the control, while at 40 and 60 mM mean growth percent differed with statistical significance from that of the control, as did all percent mean growth values with cells incubated with 3-HB. For HTB-35 cells incubated with both KBs, only the 10 mM mean % growth point did not differ significantly from that of the control. For effect of rapamycin on cells, *p*-values for all cell viabilities at each concentration of LiAcAc compared with that of the untreated control were <0.01, except for 0.5 mM and 2.0 mM concentrations of rapamycin with SW480 cells with no LiAcAc, whose values were 0.040 and 0.016.

## 3. Results

### 3.1. Sodium Acetoacetate and 3-Hydroxybutyrate Inhibit Cancer but Not Normal Cell Proliferation

**Figure 1:**
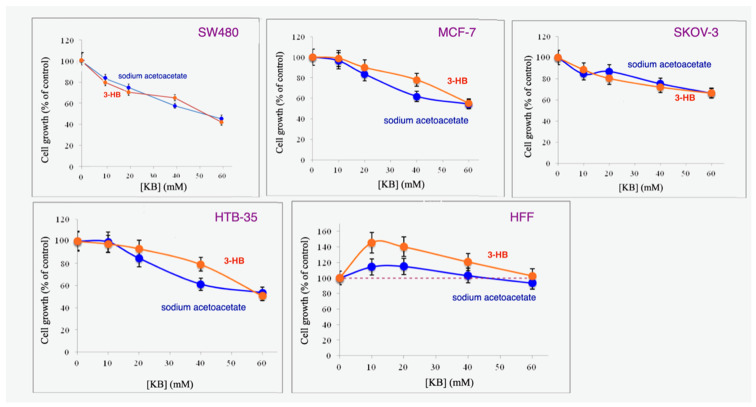
Effect of ketone bodies on cell growth. Four different cancer cell lines were incubated with sodium acetoacetate (blue) or sodium 3-hydroxybutyrate (red) and tested for cell viability using the crystal violet method for cell viability. HFF is a normal human foreskin fibroblast cell control. Statistical significance discussed in Section 2.

Figure 1 shows the dose-dependent responses of four different cancer cell lines to NaAcAc and 3-HB. Significant inhibition was achieved between 20 and 60 mM for all cells except SKOV-3 cells where the range was 40–60 mM.

For all four cancer cell lines, both agents caused approximately the same levels of inhibition. At moderate doses in cells lines MCF-7 and HTB-35, however, NaAcAc showed somewhat greater inhibition of cell growth than 3HB.

Furthermore, as in our prior studies [7,13], neither KB inhibited the growth of HFF cells from normal human fibroblasts (HFF). Inhibition of cell proliferation by ketone bodies is thus unique to cancer cells.

**Figure 2:**
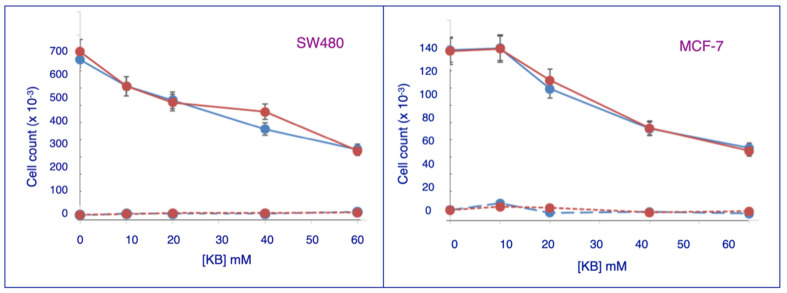
Effects of Ketone Bodies on Cell Death. The cell viability data for SW480 and MCF-7 cells from Figure 1 were replotted as live cell counts for incubation with NaAcAc (solid blue plots) or Na 3-HB (solid red plots). Concurrently, the trypan blue assay was used to detect dead cells as shown in the lower lines of both figures as dashed blue lines (NaAcAc) and dashed red lines (Na 3-HB). Initial cell counts for SW480 and MCF-7 cells were 100,000 and 50,000, respectively. The dead cell counts for both NaAcAc and 3-HB are almost horizontal lines at low cell counts, indicating that cell death for both cell lines was negligible at all concentrations of either ketone body.

To determine possible cytotoxicity, cancer cells were incubated with increasing concentrations of KB for 96 h and stained with Trypan blue. As shown in Figure 2 for SW480 and MCF-7 cells, the number of non-viable cells is virtually the same at all doses and is the same as for untreated cells (0 dose). As can be seen in the figure, cell counts for both lines remained substantially higher than the initial cell counts for the cells originally added to the wells. The initial counts for implanted cells for SW480 and MCF-7 cells were on the order of 100,000 and 50,000 respectively. At 60 mM NaAcAc, the counts were 300,000 and 60,000, respectively. Thus, both KBs inhibit cell division but do not induce cell death.

### 3.2. Effects of Lithium Acetoacetate on Cancer and Untransformed Cells

**Figure 3:**
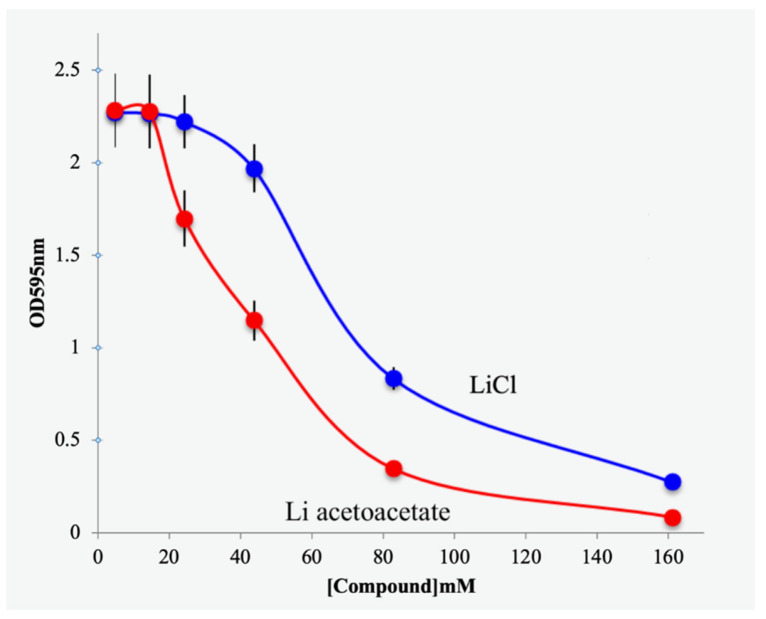
Effects of lithium ion on MCF-7 cells. Lithium chloride (blue line) or lithium acetoacetate (red line) was added MCF-7 cells and viability determined by crystal violet assay.

As noted in the Introduction, several reports have found that the inhibitory effect of lithium chloride on cancer cell growth was entirely due to the effect of lithium ion itself (as lithium acetate) [11,12]. To determine if these observations hold for MCF-7 cancer cells, we incubated cells with increasing concentrations of LiAcAc or lithium ion (as LiCl). In fact, we found that LiCl inhibited cell growth by 12% at 20 mM and approximately at 40 mM. At this concentration, NaAcAc inhibits cell growth at about 43% (Figure 1B). Thus, it appears that the effect of LiAcAc represents the combined effect of acetoacetate and lithium ions. In agreement with previous studies [11,12], lithium itself appears to be the major contributor (Figure 3).

**Figure 4:**
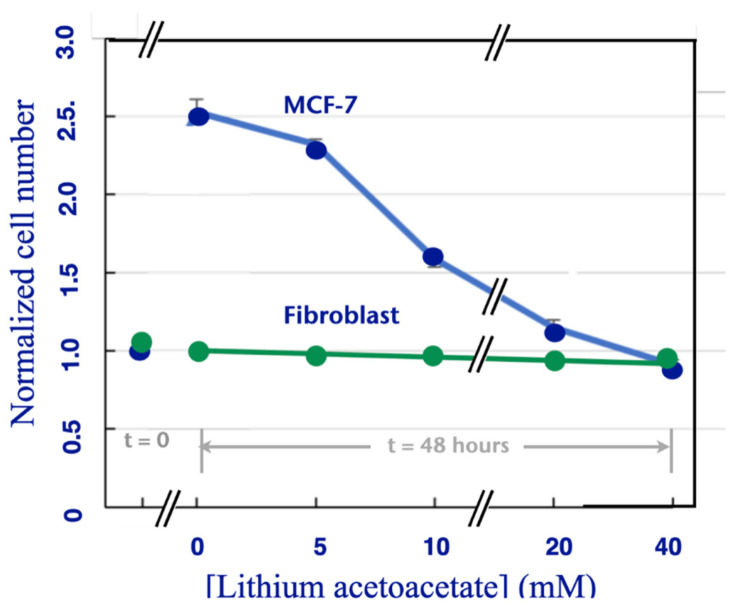
Effect of lithium acetoacetate on MCF-7 cells. Cells were incubated in the absence of LiAcAc for 48 h labeled as “0 mM” on the *x*-axis. The cells were then incubated with different concentrations of LiAcAc from 5–40 mM LiAcAc as labeled in the figure (blue line). Cell counts (*y*-axis) prior to incubation of MCF-7 cells with LiAcAc are labeled as “0 h” on the *x*-axis, i.e., the data represents the effect of lithium acetoacetate on cells that had been allowed to grow for 48 h. Controls (green line) were HSF 2617 untransformed human fibroblasts.

We determined live cell count for MCF-7 cells incubated with increasing doses of LiAcAc for 48 h. Cells were plated at a density of about 21,000 per well. As shown in Figure 4, in the absence of LiAcAc, cells proliferated to almost 60,000 per well. This proliferation number decreased as the concentration of LiAcAc increased. At a concentration of 20 mM, the cell count was close to the initial cell count for this agent, i.e., 40 mM. Trypan blue studies revealed the absence of non-viable cells. In other words, high concentrations of ketone bodies, in essence, have maintained the original population but prevented cell division. We conclude that, like its sodium analogue, LiAcAc causes inhibition of cancer cell proliferation but is not cytotoxic. Similarly, LiAcAc does not affected the growth of untransformed cells (green curve in Figure 4).

### 3.3. Effects of LiAcAc on Chemotherapeutic Agent-Induced Killing of Cancer Cells

A major aspect of the interest in ketogenic therapy and perhaps the major promise for upcoming application is the ability of ketogenic diets or agents to act as adjuvants to other modalities of treatment (reviews: [1,2,3,4,5,6]). The basic idea, again, is that if ketogenic treatment can enhance the effect of anti-cancer agents, lower doses can be used, thereby reducing side-effects and toxicity, which are characteristics and limitations of many anticancer drugs. In this, the ambiguity of lithium effects for mechanistic studies might provide a benefit in a therapeutic setting. Independent or additive effects in addition to acetoacetate itself may make lithium salt the desirable form.

We treated two different cancer cell lines, SW480 cells and MCF-7 cells, with three chemotherapeutic compounds, rapamycin, methotrexate and PNC-27, a new anti-cancer peptide [16,19], alone or in the presence of increasing doses of LiAcAc.

**Figure 5:**
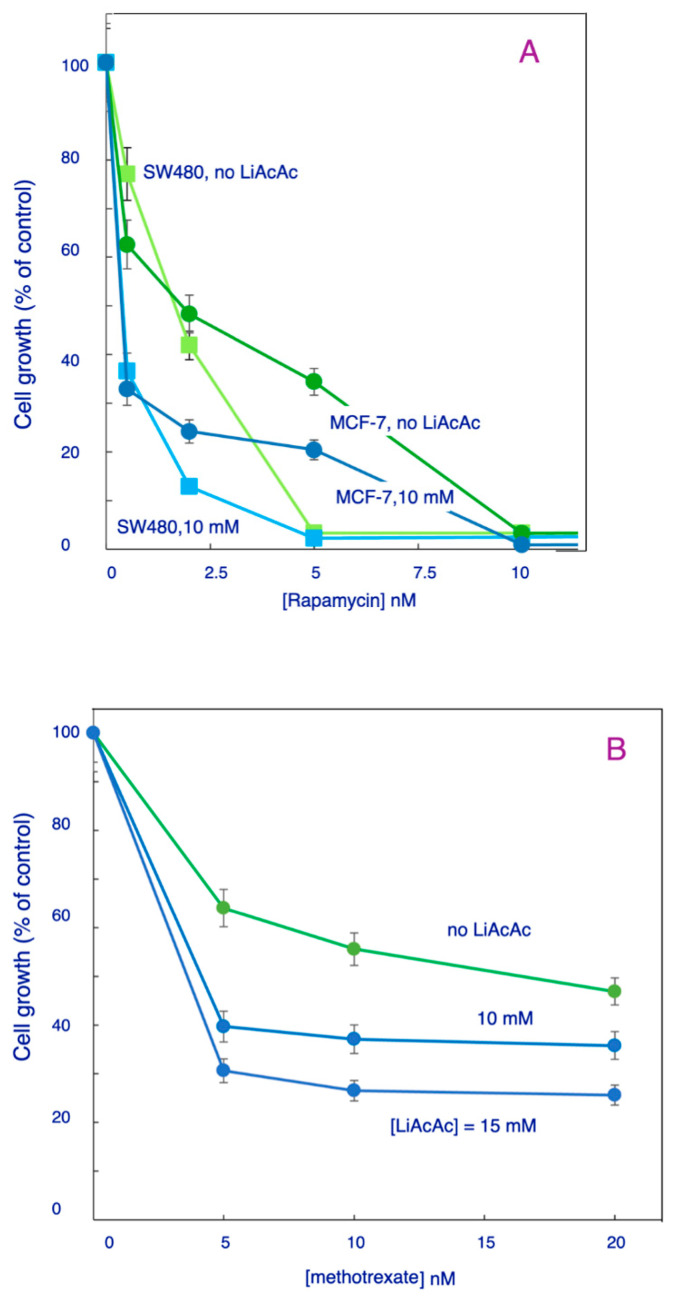

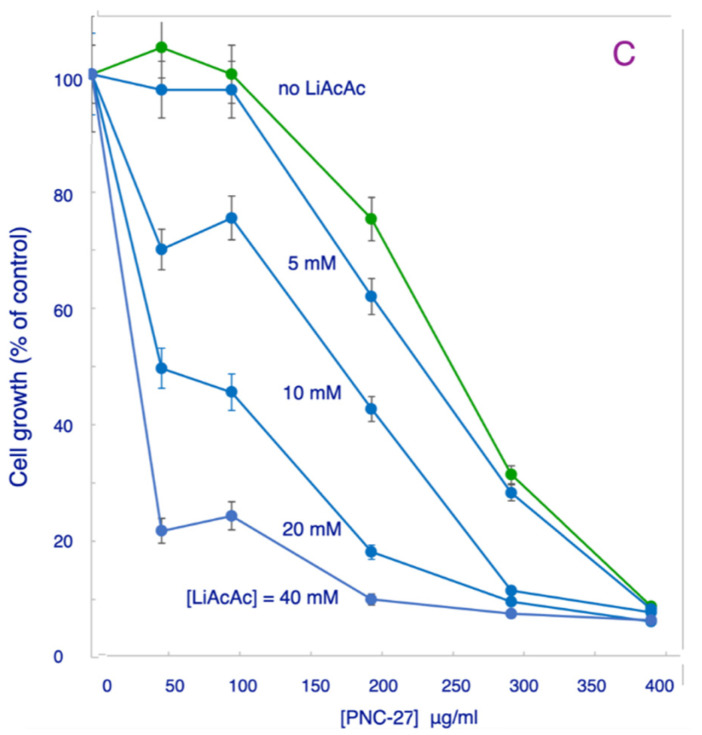
Effect of anticancer-agents on ketone body-treated cells. Two cell lines were studied: SW480 cells (square symbols in (**A**)) and (**B**) and MCF-7 cells (circles in (**A**)) and (**C**). Growth was determined with crystal violet assay. See text for details.

Figure 5A–C show the results for the effects of LiAcAc on the dose–response curves for rapamycin incubated with SW480 and MCF-7 cells (Figure 5A), methotrexate incubated with SW480 cells (Figure 5B) and PNC-27 with MCF-7 cells (Figure 5C). Figure 5A shows the results for rapamycin on SW480 and MCF-7 cells in the absence or presence of 10 mM LiAcAc. At all concentrations of LiAcAc, *p*-values for comparison of the means of triplicate assays with that of the LiAcAc-untreated controls were found to indicate significant differences, as indicated in Section 2. As can be seen in this figure, there are significant shifts of the dose–response curves to the left for both cell lines in the presence of LiAcAc. This results in a large decrease in the number of viable cells at each concentration of rapamycin. For example, at a concentration of 2 nM rapamycin, there is a decrease of about 50% of the number of viable MCF-7 cells in the presence of LiAcAc than in the absence of this KB. A similar result was observed for SW480 cells: at 2 nM rapamycin, 12 percent of cells remain viable in the presence of 10 mM LiAcAc as compared with 40 percent in its absence. As summarized in Table 1, the IC_50_ for rapamycin is reduced by LiAcAc by a factor of almost 5 for MCF-7 cells and over 4 for SW480 cells. Similar results were obtained when SW480 cells were incubated with methotrexate (Figure 5B and Table 1) LiAcAc (15 mM) reduced the IC_50_ of this agent by a factor of >2 and when MCF-7 cells were incubated with PNC-27 (Figure 5C and Table 1).

Overall, the results in Table 1 suggest that LiAcAc induces a significant reduction in the IC_50_ values for each of the three anti-cancer agents by factors ranging from 2–5. In a prior study [13], we found that NaAcAc strongly enhanced the efficacy of rapamycin on SW480 cells over a concentration range of 10–60 mM. At 40 mM NaAcAc, there was a four-fold reduction of the IC_50_ of rapamycin, in the range of 2–5. This NaAcAc concentration is four times that of the 10 mM value for LiAcAc, suggesting, as discussed above, that the latter ketone body is more effective than its sodium counterpart ketone body. This conclusion is further supported by our finding that 20 mM LiAcAc induced an almost 24-fold decrease in the IC_50_ for rapamycin-induced cell killing. This is close to the 30-fold reduction in the IC_50_ for rapamycin-induced cell killing in the presence of 60 mM NaAcAc [13].

Table 1 summarizes the effect of 10 mM LiAcAc on the killing of MCF-7 cells by PNC-27. The IC_50_ for PNC-27 when incubated with MCF-7 cells is 206 μg/mL. In the presence of 20 mM LiAcAc, the IC_50_ is 56.7 μg/mL, an almost fourfold reduction. Thus, LiAcAc is effective in reducing the IC_50_ values for a variety of chemotherapeutic agents, suggesting that ketone bodies and/or ketogenic diets may be major enhancers of anti-cancer therapy.

## 4. Discussion

### 4.1. Ketone Bodies Enhance Chemotherapeutic Molecules in Killing Cancer Cells

In our prior study [13], we found that NaAcAc and Na 3-HB inhibited the growth of SW480 colon cancer cells but did not inhibit growth of an untransformed human fibroblast (SF2617) cell line. When anti-cancer agents are incubated with cancer cells, their cell targets themselves proliferate so that the anti-cancer agents must kill the initial number of cells in culture plus the daughter cells that result from cancer cell division. We surmised that agents that can block cancer cell division, without affecting normal cells, would lower the cancer cell load on which these agents must work, thereby lowering their apparent IC_50_ values. In support of this hypothesis, we found that the IC_50_ values for rapamycin in killing SW480 cells decreased significantly with increasing concentrations of both KBs. We have therefore endeavored in this study to expand our investigation of the efficacy of KBs in lowering the IC_50_ values for anti-cancer agents, including the small molecules rapamycin and methotrexate and the anti-cancer peptide PNC-27. As we found for NaAcAc and Na 3-HB, both agents significantly lower the IC_50_ values for two small molecule therapeutic agents, rapamycin and methotrexate, using SW480 human colon cancer cells and MCF-7 breast cancer cells. In addition, LiAcAc markedly lowered the IC_50_ for PNC-27 (Figure 5C and Table 1).

A possible alternate explanation of the ketone body enhancement of chemotherapeutic agents are the effects of the ketone bodies on the distribution of cancer cells in the cell cycle. Since a number of chemotherapeutic agents have been found to act predominantly on actively dividing cells at S or G1 phases, and since resting cells may be more resistant therefore to these chemotherapeutic agents, ketone bodies might cause a shift in the cell distribution such that more cells shift from G0 to G1 or S. This would result in facilitation of chemotherapeutic agents. Since both rapamycin and methotrexate have been found to inhibit cancer cell division predominantly in S phase, ketone body facilitation might occur via this mechanism [20].

However, LiAcAc significantly lowers the IC_50_ for PNC-27 (Table 1). This anti-cancer peptide acts completely independently of the cell cycle by binding to HDM-2 which is expressed in the cancer cell membrane and forms transmembrane pores resulting in tumor cell necrosis [16]. Furthermore, if the ketone bodies were to induce shifts in cancer cell populations from resting to actively dividing, one would expect them to cause increases in cell number as a function of concentration. However, we find that the reverse occurs: the cells stop dividing as the concentration of these ketone bodies increase (Figure 1, Figure 2 and Figure 3).

### 4.2. Ketone Bodies Are Selectively Active in Blocking Cancer Cell Growth

We have found that both agents in their sodium form inhibit cancer cell proliferation but not the growth or viability of untransformed cells. Figure 1 shows that NaAcAc and Na 3-HB both inhibited the growth of four different cancer cell lines, i.e., SW480 colon cancer, MCF-7 breast cancer, SKOV-3 ovarian cancer and HTB-35 cervical cancer. This inhibition was not caused by cytotoxicity of the ketone bodies since our trypan blue exclusion studies on these KB-treated cell lines showed only minimal cell death that was present even when no KBs were present, as shown in Figure 2. This pattern was likewise found for the lithium form of AcAc, which inhibited cancer cell growth but was not cytotoxic to the cells. Importantly, we further found that NaAcAc, Na 3-HB and LiAcAc had no effect on the growth of normal or untransformed cells in culture. These results paralleled those found in an earlier study [7], discussed in the Introduction section, in which LiAcAc inhibited the growth but was non-cytotoxic to five different aggressive tumor cell lines, but did not affect the growth or viability of three untransformed cell lines. We therefore conclude that an important factor in the enhancing effect of ketogenic diets on chemotherapeutic agents on tumors in vivo is the selective blockade of tumor cell proliferation by the ketone bodies produced.

As noted in our prior study [13], the concentrations of the ketone bodies employed are non-physiological in that they would not be expected to be administered at such elevated doses as 10–15 mM LiAcAc and 20–60 mM NaAcAc and 3-HB. Importantly, these doses do not affect the viability or growth properties of normal cells (Figure 1, HF and Figure 4), suggesting that ketone bodies are non-toxic to normal cells and would not damage normal cells even at high concentrations. Rather, for normal cells, ketone bodies are metabolic substrates. As also noted in our prior study [13], the IC_50_ values for anti-tumor agents such as rapamycin and 5-fluorouracil are significantly higher in cell culture than they are as serum levels in vivo by factors of around 10. Thus it may be expected that therapeutic ranges for the ketogenic agents would be reduced around ten-fold when administered in vivo, which would further reduce the possibility of toxicity. Our previous results, showing that ketogenic diets administered to mice treated with chemotherapeutic agents for syngeneic breast cancers caused significantly increased tumor size reduction and prolonged survival [8], further suggest that administration of lower doses of ketone bodies over prolonged periods are comparable to higher doses over shorter periods.

### 4.3. Effects of Lithium and Sodium Counterions

Our results also parallel those of other studies [11,12] that found that LiAcAc inhibits cancer cell proliferation, which is associated with reduced ATP production. In contrast to our current findings, these studies also found that NaAcAc did not inhibit cancer cell growth. As noted in the Introduction section, this difference is due to the concentration ranges used in the two sets of studies, the prior studies employing LiAcAc concentrations in the 10–20 mM range, where the sodium form is only minimally active. We now find that, at higher concentrations in the 20–60 mM range, as used in this study and in our prior investigation [13], NaAcAc and Na3HB, both cause marked non-cytotoxic inhibition of cancer cell proliferation (Figure 1). Thus, LiAcAc is active at lower concentrations than NaAcAc and Na 3HB, but both forms cause significant growth arrest of cancer cells.

However, these other studies [11,12] also found that lithium in the chloride form caused the same extent of inhibition of cancer cell proliferation as the AcAc form, suggesting that lithium ion was the principal inhibitory agent. We have likewise found that, in MCF-7 breast cancer cell lines, LiCl inhibits proliferation of these cells but that LiAcAc inhibits their proliferation to a greater extent, as shown in Figure 3, suggesting that AcAc does play a major role in cancer cell proliferation inhibition. As shown in Figure 3, there appears to be a constant increase in the level of inhibition of cancer cell growth by LiAcAc compared with that of LiCl, i.e., the inhibition curves are parallel for the two agents.

In addition, in the prior studies with LiCl and LiAcAc, both agents were found to cause the decreased proliferation of untransformed cells [11,12]. As shown in Figure 1, and in our prior study [13], the sodium forms of both AcAc and 3-HB did not inhibit the growth of untransformed human fibroblasts (Figure 1 HFF). Also, LiAcAc inhibited cell proliferation of MCF-7 cells but did not induce cell death (Figure 4). These results with LiAcAc are consistent with our prior findings on five different cancer cell lines and three untransformed human cell lines [7].

Possible reasons for these different observations may be related to experimental conditions and/or to properties of lithium. In our experimental protocol, cells are incubated in media containing different concentrations of ketone bodies for 96 h while, in the other prior studies, there is an initial incubation period of 24 h in media, followed by a 48 h incubation in media with a ketone body at a given concentration, followed by another 24 h incubation in identical fresh ketone body-containing media. This procedure results in an exposure time of the cells to ketone bodies of 72 h rather than 96 h as in our protocol. In our prior study [13], we found that large increases in inhibition of cell proliferation occur in the 48–96 h time period, so that time of exposure may be a possible significant difference, although this does not explain the difference in our findings that both KBs do not affect normal cells.

### 4.4. Possible Effects of Lithium Ion on Cancer Cell Growth

Another possible cause for differences in results concerning the effects of LiAcAc may be related to the differences in cell lines used in the various studies [10]. Lithium ion has been found to have different effects on the proliferation of different cancer cell lines [10]. These differences are related to the effects of lithium on different signal transduction pathways in cells. Depending on which pathway is affected, cells may be induced to undergo apoptosis or increased proliferation. At the same time, several critical targets of lithium appear to have dual contradictory functions (e.g., apoptosis vs cell proliferation). For example, a major target of lithium inhibition is the enzyme glycogen synthase kinase 3β (GSK-3β), which inhibits the Wnt/β-catinin pathway involved in proliferation but concurrently also activates the NFκB -induced pathway that promotes cell proliferation [10]. Depending on which of the two pathways becomes dominant, cells will either undergo apoptosis or proliferation. In neuroblastoma cells, lithium activates GSK-3β that has been found to activate p53 that induces apoptosis of these cells [10]. On the other hand, in HepG2 hepatoblastoma cells, lithium inhibition of GSK-3β results in blockade of its interaction with p53 resulting in decreased apoptosis and increased cell proliferation [10]. In some cell lines (MK3 and MK4), the effect of lithium on proliferation is dose-dependent, such that doses of 10–40 mM stimulate proliferation, while at 50 mM or higher, proliferation is inhibited [10]. Interestingly, of the multiple studies on lithium effects on cell proliferation, few have focused on its effects on metabolic pathways, such as glycolysis, Krebs Cycle, Randle Cycle, etc. One study suggested that lithium ion induced the detachment of hexokinase from the mitochondrial membrane of B16 mouse melanoma cells resulting in inhibition of cell proliferation [21].

Including our present study and our past study [7] on the effects of LiAcAc on cancer and normal cell growth, we have used three colon cancer cell lines (CACO2, SW48 and SW480), two breast cancer cell lines (MDA MB 231 and MCF-7), one ovarian cancer cell line (SKOV-3) and one cervical squamous carcinoma cell line (HTB-35) and multiple different normal human fibroblast cell lines, including RFP3, MCH064, MCH065, HSF2617 and HFF. In one of the other studies [11], three breast cancer cell lines (MCF-7, MDA MB 231 and Hs578T) and one normal fibroblast line (HSF064) were used and, in the other study [12], two neuroblastoma cell lines [SH-SY5Y and SK-N-BE(2)], two renal cell carcinoma cell lines (786-O and CAKI-2) and two normal control cell lines [HEK-293 embryonic renal cells and HDFn (normal dermal fibroblasts) were used. The only overlap in cell lines that were common to our study were MDA MB 231 and MCF-7 cells in one [11] of the two other studies. It is possible that, given the variable effects of lithium on different cell lines and even its variable effects on the same cell lines depending on dose, different results using lithium salts can be obtained.

In view of our findings that Na and Li AcAc, and Na 3OH, cause non-cytotoxic inhibition of cancer cell proliferation and do not appear to affect the growth of normal cells and significantly lower the IC_50_ values for three different small molecule chemotherapeutic agents, they appear to be excellent candidates for adjuvant therapy in treating human tumors.

## 5. Conclusions

We summarize our findings in this study as follows:

Figure 1 shows that increasing concentrations of NaAcAc and 3-HB cause a statistically significant, dose-dependent decrease in cell proliferation. Neither ketone body inhibits cell growth of normal cells (Figure 1-HFF).

Both ketone bodies cause dose-dependent decrease in cell proliferation in cancer lines SW480 and MCF-7 (Figure 2). Treatment with ketone bodies does not induce cell death, as shown in the trypan blue results.

Based on these two sets of findings we conclude that the ketone bodies selectively block cancer cell growth but do not affect normal cell growth.

We further find that lithium acetoacetate (LiAcAc) inhibits cell proliferation at lower concentrations than the corresponding sodium salt (Figure 3). The dose-dependent inhibition of cell growth caused by LiAcAc appears to be due to the combined effects of Li ion (as LiCl) and AcAc.

NaAcAc and LiAcAc facilitate the action of chemotherapeutic agents by causing a significant lowering of the IC_50_ of these agents as indicated by the effect of this rapamycin-induced killing of SW480 and MCF-7 cells. Table 1 indicates the significant factors by which LiAcAc and NaAcAc lower the IC_50_ values in these cell lines.

## Figures and Tables

**Table 1:** Reduction of the IC_50_ Values for Chemotherapeutic Agents on Two Cancer Cell Lines by LiAcAc.

Cell Line	Ketone Body	Chemotherapeutic Agent	IC_50_	IC_50_ Ratio ^1^
SW480	None	Rapamycin	1.655 nM	
SW480	10 mM LiAcAc	Rapamycin	0.394 nM	4.2
SW480	20 mM LiAcAc	Rapamycin	0.0692 nM	23.94
SW480	None	Methotrexate	0.1965 nM	
SW480	15 mM LiAcAc	Methotrexate	0.0863 nM	2.28
MCF-7	None	Rapamycin	1.822 nM	
MCF-7	10 mM LiAcAc	Rapamycin	0.368 nM	4.95
MCF-7	None	PNC-27	207 μg/mL	
MCF-7	20 mM LiAcAc	PNC-27	56.7 μg/mL	3.65

^1^ Ratio of the IC_50_ for the chemotherapeutic agent in the absence of LiAcAc to the IC_50_ for the chemotherapeutic agent in the presence of the specified concentration of LiAcAc.

## Data Availability

All data is available by request from the principal author.

## Abbreviations

Li, lithium; AcAc, acetoacetate; 3-HB, 3-hydroxybutyrate; KB, ketone bodies, i.e., AcAc and 3-HB.

## References

[1] Champ C.E., Palmer J.D., Volek J.S., Werner-Wasik M., Andrews D.W., Evans J.J., Glass J., Kim L., Shi W. (2014). Targeting metabolism with a ketogenic diet during the treatment of glioblastoma multiforme. J. Neurooncol..

[2] Tan-Shalaby J. (2017). Ketogenic Diets and Cancer, Emerging Evidence. Fed. Pract..

[3] Klement R.J. (2017). Beneficial Effects of Ketogenic Diets for Cancer Patients: A Realist Review with Focus on Evidence and Confirmation. Med. Oncol..

[4] Seyfried T.N., Kiebish M., Mukherjee P., Marsh J. (2008). Targeting energy metabolism in brain cancer with calorically restricted ketogenic diets. Epilepsia.

[5] Poff A.M., Ari C., Seyfried T.N., D’Agostino D.P. (2013). The Ketogenic Diet and Hyperbaric Oxygen Therapy Prolong Survival in Mice with Systemic Metastatic Cancer. PLoS ONE.

[6] Fine E.J., Segal-Isaacson C.J., Feinman R.D., Herszkopf S., Romano M.C., Tomuta N., Bontempo A.F., Negassa A., Sparano J.A. (2012). Targeting Insulin Inhibition as a Metabolic Therapy in Advanced Cancer: A Pilot Safety and Feasibility Dietary Trial in 10 Patients. Nutrition.

[7] Fine E.J., Miller A., Quadros E.V., Sequeira J.M., Feinman R.D. (2009). Acetoacetate Reduces Growth and ATP Concentration in Cancer Cell Lines Which Over-Express Uncoupling Protein 2. Cancer Cell Int..

[8] Zou Y., Fineberg S., Pearlman A., Feinman R.D., Fine E.J. (2020). The Effect of a Ketogenic Diet and Synergy with Rapamycin in a Mouse Model of Breast Cancer. PLoS ONE.

[9] Warburg O. (1956). On the Origin of Cancer Cells. Science.

[10] Villegas-Vázquez E.Y., Quintas-Granados L.I., Cortes H., González-Del Carmen M., Leyva-Gómez G., Rodríguez-Morales M., Bustamante-Montes L.P., Silva-Adaya D., Pérez-Plasencia C., Jacobo-Herrera N. (2023). Lithium: A Promising Anti-Cancer Agent. Life.

[11] Cohen-Harazi R., Hofmann S., Kogan V., Fulman-Levy H., Abaev K., Shovman O., Brider T., Koman I. (2020). Cytotoxicity of Exogenous Acetoacetate in Lithium Salt Form Is Mediated by Lithium and Not Acetoacetate. Anticancer. Res..

[12] Vidali S., Aminzadeh-Gohari S., Vatrinet R., Iommarini L., Porcelli A.M., Kofler B., Feichtinger R.G. (2019). Lithium And Not Acetoacetate Influences the Growth of Cells Treated with Lithium Acetoacetate. Int. J. Mol. Sci..

[13] Miller A., Lin B., Pincus M.R., Fine E., Feinman R.D. (2021). Selective Enhancement of Chemotherapeutic Agent-Induced Tumor Cell Killing by Acetoacetate and 3-Hydroxybutyrate. EC Pharmacol. Toxicol..

[14] López-Soriano F.J., Argiles J.M. (1985). A Simple Method for the Preparation of Acetoacetate. Anal. Lett..

[15] Schilke R.E., Johnson R.E. (1965). A Colorimetric Method for Estimating Acetoacetate. Am. J. Clin. Pathol..

[16] Wang H., Zhao D., Nguyen L.X., Wu H., Li L., Dong D., Troadec E., Zhu Y., Hoang D.H., Stein A.S. (2020). Targeting cell membrane HDM2: A novel therapeutic approach for acute myeloid leukemia. Leukemia.

[17] Feoktistova M., Geserick P., Leverkus M. (2016). Crystal Violet Assay for Determining Viability of Cultured Cells. Cold Spring Harb. Protoc..

[18] Strober W. (2016). Trypan Blue Exclusion Test of Cell Viability. Curr. Protoc. Immunol..

[19] Sarafraz-Yazdi E., Mumin S., Cheung D., Fridman D., Lin B., Wong L., Rosal R., Rudolph R., Frenkel M., Thadi A. (2022). PNC-27, a Chimeric p53-Penetratin Peptide Binds to HDM-2 in a p53 Peptide-like Structure, Induces Selective Membrane-Pore Formation and Leads to Cancer Cell Lysis. Biomedicines.

[20] Sun Y., Liu Y., Ma X., Hu H. (2021). The Influence of Cell Cycle Regulation on Chemotherapy. Int. J. Mol. Sci..

[21] Penso J., Beitner R. (2003). Lithium Detaches Hexokinase from Mitochondria and Inhibits Proliferation of B16 Melanoma Cells. Mol. Genet. Metab..

